# The “Yellow Fever Plot”: Biological Warfare in the American Civil War

**DOI:** 10.7759/cureus.106645

**Published:** 2026-04-08

**Authors:** Matthew D Turner

**Affiliations:** 1 Emergency Medicine, Penn State Health Milton S. Hershey Medical Center, Hershey, USA

**Keywords:** american civil war, biological warfare, bioterrorism, malaria, medicine history, smallpox, yellow fever

## Abstract

The American Civil War was unprecedented in both its brutality and bloodshed. Disease and the threat of biological warfare played a key role in the conflict, most notably in a Confederate plot to create an epidemic of yellow fever in the Union. Other instances of biological warfare included the Confederate’s hopes that endemic diseases would offer a military advantage, the poisoning of wells, accusations of the deliberate spread of smallpox, and even unsubstantiated rumors of entomological warfare. In this narrative review, we examine the role that biological warfare played in the American Civil War.

## Introduction and background

The American Civil War, ranging from 1861 to 1865, was the most devastating event that the United States has ever experienced. The conflict, which saw the deployment of vast armies, new military innovations, and atrocious casualties, has reverberations upon the United States to this very day [1]. The degree that biological warfare, defined as the use of biological agents such as insects, bacteria, and other pathogens [2], had upon the American Civil War remains a relatively obscure subject, despite the vast influence that disease had upon the war’s outcome.

While modern germ theory had not yet been developed during the American Civil War, it should be noted that contemporary physicians had a vastly improved understanding of disease, particularly in a wartime setting, than in prior conflicts. In the 1846-1848 Mexican-American War, there had been 7-10 deaths from disease for every death in combat [3], and in the 1853-1856 Crimean War, there had been four deaths from disease for every death in combat [4]. In the 1861-1865 American Civil War, there were only two deaths from disease for every one death in combat [3]. Even so, one 1993 estimate determined that infectious disease likely prolonged the war by an additional two years [5].

In this context, military planners of both sides took disease into account while planning the war. This primitive biological warfare was largely designed to take advantage of, or overcome, the endemic diseases of the South, but it also extended to mutual accusations of deliberate spread of disease, as well as one of the first bioterrorism plots in modern history.

In this narrative review of the limited literature, we strive to examine the historical evidence for biological warfare during the American Civil War and the implications that it had on the conflict.

## Review

A brief introduction to biological warfare

For the vast majority of human history, disease has had a far greater impact on the number of soldiers killed in war than in combat [6]. Armies have recognized the impact of disease on warfare since ancient times, and so biological warfare may be understood as part of traditional warfare, with a history that extends for thousands of years [7]. Biological warfare may be understood as the use of toxins, microorganisms, and various pathogens to deliberately target enemy soldiers, infrastructure, and agriculture. The poisoning of wells with dead animals or manure [6], the deliberate catapulting of dead plague victims, manure, and cattle into besieged cities [6], the use of ​​​​​​arrows contaminated with *Clostridium tetani* by Melanesian tribesmen [2], and the deliberate spread of smallpox-contaminated blankets by Sir Jeffrey Amherst to Native American tribes in 1763 [2] are a mere handful of examples predating the American Civil War.

By the time of the American Civil War, the concept of biological warfare was common across North America. Amherst’s attempt to spread smallpox-contaminated blankets to Native Americans was well-known, and rumors of other deliberate attempts to spread smallpox to Native American tribes, such as a 1770 Ojibwa account of traders spreading smallpox through deliberately contaminated flags, were widespread [8]. Interestingly, the British also accused Native Americans of practicing biological warfare; during Queen Anne’s War, a group of Iroquois hunters reportedly dumped a large number of animal carcasses into a small creek upstream of an encamped British army. The unsuspecting British drank from the water and reportedly suffered over a thousand casualties [8]. The British also suspected the French of deliberately releasing smallpox-infected prisoners into Nova Scotia in 1757 [8]; likewise, rumors persisted among Patriot forces that the British were deliberately trying to infect the Continental Army with smallpox during the American Revolution [9]. Regardless of the veracity of these stories, they demonstrate that biological warfare was a well-established fear within the region by the time of the American Civil War.

Case study: The Yellow Fever Plot

**Figure 1:**
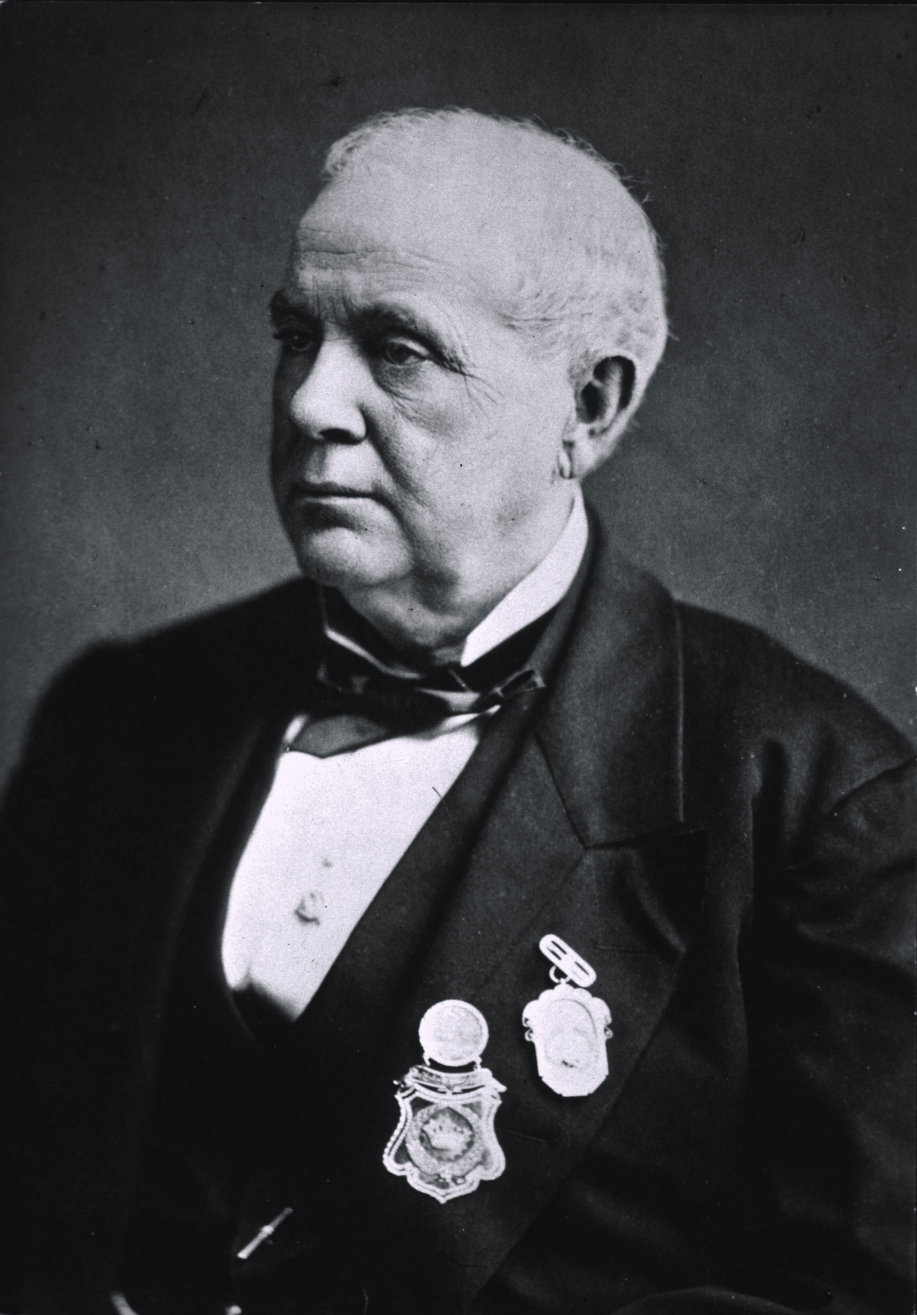
Dr. Luke Blackburn This picture is from the National Library of Medicine and is in the public domain [12].

Dr. Luke Pryor Blackburn (Figure 1), a middle-aged physician from Kentucky, was an ardent supporter of the Confederacy. He had a long fascination with infectious diseases, particularly yellow fever [10]. “Yellow Jack,” as discussed later, was endemic to the southern United States at the time and regularly decimated coastal cities. Blackburn’s efforts at controlling and preventing yellow fever outbreaks, in locales as varied as New Orleans, Kentucky, and Bermuda, had been quite successful, thanks to his emphasis on sanitation and quarantines [10]. He achieved international acclaim for his brilliant work in treating yellow fever and had even been awarded 100 pounds and a special commendation by Queen Victoria of Great Britain [11].

During the war, Blackburn spent much of his time in Montreal and Toronto, two Canadian cities that were hotbeds for Confederate sympathizers. Blackburn, a Confederate supporter, was well-connected in these circles; in one October 18, 1864, meeting, he is listed as meeting with a number of other Confederate supporters, including John Wilkes Booth, the future assassin of President Abraham Lincoln [10]. Blackburn soon became interested in the idea of employing biological weapons against the Union population and promised that he would create “an infallible plan directed against the masses of Northern people solely to create death” [11]. Blackburn was not the first Confederate to come up with the idea of biological warfare. Several years earlier, a farmer named R.R. Barrow proposed spreading clothing from those who had died from yellow fever among the Union garrison in New Orleans to cause a deadly outbreak [13]. Blackburn’s plan was similar, but much grander in scale, and, unlike Barrow, Blackburn proceeded far beyond the planning stages.

In the spring of 1864, he learned of a fresh outbreak of yellow fever in Bermuda. Determined to obtain material for a biological attack, the physician traveled to the island, where he began to collect the clothes and bedsheets of the sick and dying. One of his nurses later testified that the physician ordered her to cover sick and feverish patients with piles of clothing. Once the clothing was soaked with the sweat and vomit of the patient, he would then collect the contaminated clothing in a trunk for his own personal use [14].

Blackburn eventually collected several trunks filled with contaminated clothing and brought them along when he returned to Canada. There, he hired Godfrey Joseph Hyams, an Englishman sympathetic to the Confederate cause. With the promise of a $100,000 award, Hyams would smuggle the trunks across multiple Union cities, including Philadelphia and Washington, DC. From there, Blackburn hoped that the contaminated clothes would trigger an outbreak of yellow fever across the north, bringing the Union war effort to a grinding halt [11]. One of his targets was the White House; he had a special trunk prepared with a number of ornate dress shirts intended for President Lincoln [11]. Several months later, Blackburn returned to Bermuda, where he again collected trunks of contaminated clothing [11].

Rumors of Blackburn’s unusual activities, as well as his ardent support for the Confederacy, attracted the attention of the US consul in Bermuda, who began to investigate the physician’s behavior. Meanwhile, Blackburn’s failure to obtain funding for Hyams caused the Englishman to abandon the plot. In April 1865, Hyams went to Union authorities and revealed the plan. He notably claimed that he had spread a number of the trunks to Union cities and that some of the dress shirts intended for Lincoln had made it to the White House, although there is no evidence to support this claim [11]. Investigating Hyams’ story, authorities in Bermuda found a number of the trunks, still filled with stained and contaminated clothing [11]. The news broke to the shocked public in May 1865, when the New York Times published an article on the so-called “Yellow Fever Plot” [14].

Despite his newfound infamy, Blackburn was never arrested in the United States; instead, he was arrested and tried in Canada for violating its neutrality status. He would not return to the United States until 1873. Strangely enough, he served a term as Kentucky’s governor from 1879 to 1883. He died in 1887, nicknamed “The Good Samaritan” by his constituents [10].

While Blackburn was successful in obtaining contaminated clothing from the outbreak, he was limited by a fundamental misunderstanding of yellow fever; instead of being transmitted by fomites like smallpox, yellow fever is transmitted through mosquito bites. This mode of transmission would not be discovered until 1900 [2]. There is some evidence to suggest that Blackburn originally considered using smallpox-contaminated clothing as an alternative tactic; had he chosen this pathogen, the outcome of the plot could have been devastating [13]. A similar tactic was reportedly used by Lord Jeffrey Amherst at Fort Pitt in 1763, when his soldiers distributed smallpox-contaminated blankets to non-immune Native American tribes, unleashing a deadly outbreak [15].

Blackburn’s plot was also limited by its crude dissemination plan, which hinged upon a single unreliable agent that ultimately revealed the scheme. This may have been due to a simple lack of resources. It remains unclear if Blackburn was acting alone or to what degree the Confederate government supported him; there is some evidence to suggest that the “Yellow Fever Plot” had the explicit support and financial backing of Confederate President Jefferson Davis [16]. However, regardless of its inherent flaws, Dr. Blackburn’s “Yellow Fever Plot” should be regarded as one of the most dangerously innovative attempts at biological warfare of its time.

An Aberration or a Precursor?

Although Blackburn’s plot, based on an incorrect understanding of the transmission of yellow fever, was fundamentally flawed, it bears an uncanny resemblance to the use of biological weapons by Rhodesia during the Rhodesian Bush War of 1964-1979. Although the Confederate and Rhodesian regimes existed on different continents, over a century apart, both regimes shared a number of traits that have often led to the deployment of otherwise taboo weaponry: both Confederate and Rhodesian forces were desperately short on resources, engaged in civil war against a numerically superior enemy, facing the imminent end of their respective regime’s survival, and isolated diplomatically, economically, and politically on the world stage. As Cross identifies in his superb work on the Rhodesian chemical and biological weapons (CBW) program, these factors (forcing an isolated, crumbling power into extremis) often lead desperate regimes to employ weapons that would be otherwise unthinkable in a conventional environment [17].

Within this framework, Blackburn’s attempt at biological warfare, while haphazard and doomed from the start, can be viewed not as an unusual footnote in the American Civil War but as a precursor to the biological warfare programs of later regimes. Similar conflicts in the future may descend into similar barbarity.

Role of endemic diseases

In the early days of the war, physicians on both sides of the conflict predicted that the twin endemic diseases of the South (yellow fever and malaria) could potentially give a decisive advantage to the Confederacy [4]. Indeed, the “sickly season” of the South, typically between the summer and fall, when these diseases were at their highest prevalence, was a source of great concern for many Union commanders [18]. Winfield Scott, Commanding General of the US Army, specifically called for any invasions of the Confederacy to take place after November to be “after the return of frosts to kill the virus of malignant fevers below Memphis” [19]. This initially gave the Confederacy, largely fighting a defensive war, an advantage: during the “sickly season,” Southerners could reasonably expect Union forces to be wary of launching any major offenses [18]. However, Southern hopes that endemic disease would provide a decisive advantage in the war soon proved to be a mirage.

Yellow Fever

Yellow fever is a *Flavivirus* virus transmitted by arthropods, most often the *Aedes aegypti* mosquito. First documented in 1648, it routinely devastated North American cities up to the early 20th century [20]. Symptoms range from nonspecific fever, chills, and general malaise to severe sepsis with anywhere from 20%-50% mortality rates [21].

In the South, yellow fever was sometimes called the “stranger’s disease” as it often affected newcomers to an area, as those who survived it had lifelong immunity [3]. It was most often seen in southern ports, particularly New Orleans, widely considered one of the unhealthiest places in the country to live. Outbreaks of the dreaded “yellow jack” swept through the port with terrifying frequency: the city saw outbreaks in 1853, 1854, 1855, and 1858, the most recent of which had utterly decimated the non-immune immigrant population [18]. Southerners were generally considered to be immune to the disease. As yellow fever is primarily transmitted via the *Aedes aegypti* mosquito, which is extremely sensitive to cooler temperatures, much of the Northern population had never been exposed to the disease and were thus more vulnerable [22].

When Union forces occupied New Orleans in 1862, Confederate forces eagerly predicted that yellow fever would kill thousands of the occupying force [4]. Confederate Major General Mansfield Lovell took this anticipated outbreak into consideration, declaring that he would use a force of five thousand men to keep Union forces pinned within New Orleans, and “thus subject [them] to the diseases incident to that city in summer” [18]. Even within the city, it was rumored that locals were “intentionally creating unsanitary conditions in the city in order to increase the likelihood of an epidemic” [18].

Confederate confidence in yellow fever correlated with Union fears. Yellow fever was a source of great nervousness for Union forces, seeming to be a “terrifying and mysterious southern disease that could appear out of thin air at any moment and wipe out an unacclimated brigade, division, or crew in a matter of weeks” [18]. Major General Benjamin E. Butler, in charge of the occupation of New Orleans, was determined to prevent an outbreak. To his disgust, a number of his officers requested transfers or tried to resign their commands when they found out that they would be occupying New Orleans, the epicenter of the dreaded “Yellow Jack” [18].

Butler pursued an aggressive strategy that incorporated both rigorous sanitation standards for the city, incorporating an army of laborers to clean, regular inspections, new technology such as steam-powered pumps to siphon away stagnant water, and a harsh quarantine system. Any ships headed to the city were stopped 70 miles south of the city. Those suspected of containing yellow fever were held in quarantine for 40 days [18]. He assembled a team of military and local physicians and gave one of them the following orders: “In this matter, your orders shall be absolute … so long as you keep yellow fever away from New Orleans, your salary shall be one thousand dollars per month. When yellow fever appears in this city, your pay shall cease” [3].

Butler’s strategy for managing yellow fever was remarkably successful. Despite Confederate hopes, only two yellow fever deaths occurred in all of 1862 [18]. Throughout the entire war, only 11 victims died of yellow fever during the Union occupation [22]. The city would not experience any subsequent outbreaks until 1873, when occupying troops had long since departed [4].

While there were a number of outbreaks of yellow fever at other Union garrisons across the South [18], with one notable epidemic occurring in Wilmington in 1862 [22], the disease ultimately had a minimal impact on the war. In a postwar report entitled *The Medical and Surgical History of the War of the Rebellion*, Dr. Joseph Woodward determined that there had only been 1,355 cases of yellow fever and 436 deaths from it across all Union forces throughout the war [18]. One of the great scourges of the South, expected to be a powerful weapon against the Union, had been defeated.

Malaria

Malaria is a vector-borne disease caused by *Plasmodium *parasites, transmitted by mosquitoes. It is most often characterized by periodic fevers and may cause relapses for months or even years after the initial infection [23]. The impact that malaria could have on military campaigns had been known for centuries prior to the American Civil War; it had played a decisive role in the Haitian Revolution, and over half a century earlier, Napoleon Bonaparte had deliberately flooded entire regions with brackish water to cause an outbreak of malaria in invading British forces, declaring, “We must oppose the English with nothing but fever, which will soon devour them all” [24].

Malaria, also called “intermittent fever” or “periodic fever” [25], was endemic to the South during the war. Largely of the *Plasmodium falciparum* variety, it was most often spread by the *Anopheles quadrimaculatus* mosquito. Contemporary physicians attributed malaria to the putrid air emitted by swamps, stagnant water, and rotting vegetation, most often found in the humid coastal regions of the South. They were not entirely incorrect: such environments overwhelmingly favored mosquito reproduction [22]. Malaria was easily recognized by the periodic fever, chills, sweats, and nausea followed by periods of recovery and relapses that could last for years [22].

Repeated malarial infections may lead to partial immunity, where reinfection will only produce minimal symptoms [22]. Just as with yellow fever, Confederates hoped that malaria would incapacitate invading Union armies. One physician wrote, “… it was asserted that from the James and the Savannah to the Mississippi and the Gulf, unacclimated soldiers would quickly be disabled by the pernicious malarial fevers that prevailed over more than half the region and, for nearly half the year, south of the Ohio…” [4].

Southern forces took advantage of malaria during the early days of the war. In the 1862 Peninsular Campaign, Confederate General Joseph E. Johnston effectively used his smaller force to force the larger Army of the Potomac near the Chickahominy River, a swampy region endemic with malaria due to its massive mosquito population. Barely five miles from the Confederate capital, the Union forces were devastated by the disease. Hampered by mounting medical casualties, the Army of the Potomac was bogged down and finally forced to retreat [19]. A similar outcome occurred in the West when, in 1863, the Union Army of the Arkansas was bogged down by endemic malaria as it advanced down the Mississippi River, massively hindering its effectiveness [19]. Multiple other Union regiments would be severely hampered by malaria during the war [4].

However, the Confederacy failed to anticipate the impact of quinine upon the war. The bark of the cinchona tree in South America has been used to treat malaria for centuries. The 19th-century identification of quinine, the alkaloid responsible for this effective treatment, revolutionized malaria treatment [26]. By the time of the American Civil War, quinine was the drug of choice for treating malaria [25]. A short-acting medication, it was often distributed in salt form and would be dissolved in a drink [27], typically whiskey for Union forces [28]. Modern readers may recognize it as the “bitters” taste in gin and tonic [27]. While it was an imperfect drug with a number of unpleasant side effects, a 1866-1868 study conducted after the war found that quinine had a cure rate of over 98% [26]. Union forces rapidly embraced it [4].

Unfortunately for the Confederacy, the Union blockade effectively choked off the rebellion’s quinine supply. Confederate manpower was crippled by recurrent bouts of malaria, steadily reducing the effectiveness of their steadily depleted forces [25]. One report estimated that malaria was responsible for over 115,000 cases of sickness in the Confederate army for 1861 and 1862 alone [25]. Southern prices of quinine sulfate rose from $5 an ounce to $500 an ounce by the war’s end [19].

Confederate forces tried to alleviate this critical shortage through several strategies. Blockade runners and smugglers were unable to provide an effective supply [25], although the opportunities were lucrative; one smuggler reportedly snuck in $10,000 worth of quinine hidden within the corpse of a mule [19]. The growing shortage of quinine was a source of consternation for the public, and in some areas, it cost the Confederate government valuable political support [18]. Meanwhile, Confederate physicians desperately tried to find an alternative antimalarial treatment, testing dozens of indigenous plants. Testing was inconsistent, limited by both the Confederacy’s critical shortage of resources and by its climate, as stored medicines often lost their potency due to the higher temperatures of the South [25].

In contrast, Union forces had a relatively plentiful supply of quinine, consuming approximately 19 tons of quinine throughout the conflict [19]. It was regularly distributed in prophylactic form and significantly reduced mortality among Union forces [4]. A number of historians have argued that quinine was one of the most powerful weapons in the Union arsenal [18]. As the war dragged on, Union hospitals also came to use “mosquito bars,” expensive mosquito netting that further reduced cases [22]. It was also a common observation that blacks were less vulnerable to malaria than whites, and so Union forces also tended to assign black troops to areas that were most affected by malaria, such as the coastline of the Carolinas and the lower Mississippi River [4]. The Confederacy, fighting to preserve the institution of race-based slavery, had no such option. By the war’s end, contrary to Confederate expectations, malaria was far more devastating to Confederate manpower than to the Union [28]. Just as with yellow fever, the “idea that the fevered Southern environment would repel the white strangers wearing blue did not help the Southern cause” [4].

Other accusations of biological warfare

Well Poisonings

Biological warfare via the poisoning of water supplies, often by leaving dead animal carcasses in wells or ponds, is a form of biological warfare that has been documented for millennia [29]. In 1863, as Confederate forces retreated from Vicksburg, they deliberately contaminated water supplies with animal corpses [13]. In response, the Union War Department issued the following command in General Orders No. 100 in April, stating, “The use of poison in any manner, be it to poison wells, or food, or arms, is wholly excluded from modern warfare” [30].

Accusations of Deliberate Disease Spread in Prisoners

Despite some initial prisoner exchanges, by 1863, the system of prisoner exchange between the two sides had broken down, and both the Union and the Confederacy began to construct massive prisoner-of-war (POW) complexes [4]. The conditions found within these sprawling, overcrowded, and poorly planned camps, in which over 400,000 soldiers had been contained by the war’s end, were utterly deplorable. Starvation, minimal medical care, and poor sanitation were endemic. One in seven POWs would not survive the experience [31]. Places such as the Confederate prison of Andersonville became legendary for the horrors they contained [31].

Both sides accused the other of deliberately making POW camps as inhospitable as possible to deliberately sicken captives. Southern troops, often from more rural areas and less likely to be vaccinated than their northern counterparts, were especially vulnerable to smallpox, which often ravaged the camps [4]. The Confederate government attempted to address this low vaccination rate during the Civil War, ultimately to little success [32]. In contrast, the Union had a much more robust system of vaccination for its citizens and soldiers [32].

A number of Confederates claimed that the Union had deliberately spread smallpox among Confederate prisons, particularly prisoners due to be released. In one notable prisoner swap in 1862, 3 out of 202 released Confederates were found to be ill with smallpox. General P.G.T. Beauregard, commander of the local Confederate forces, angrily wrote that “… [this] looks very much like an attempt to communicate the small-pox to my command” [4]. While the released prisoners were quarantined and no significant outbreak occurred, suspicions would linger for the rest of the war. A minor outbreak of smallpox occurred in the Confederate Army of Northern Virginia from 1862 to 1864, again triggering accusations that the Union deliberately released infected prisoners into the wider Confederate population [4]. However, there is no evidence to back up these claims; it is far more likely that this was due to the Army of Northern Virginia’s unexposed troops spending more time in smallpox-endemic areas of Maryland [4]. Likewise, outbreaks of smallpox among Confederate POWs can be attributed to the cheap and rushed construction of POW camps [31]. It should be noted that men such as Union general Benjamin Butler went out of their way to send smallpox vaccines across the battle lines to the South [31].

Entomological Warfare During the American Civil War

Entomological warfare is a subset of biological warfare, employing the use of insects as weapons of war, whether as direct stinging/irritating weapons, as disease vectors, or even as a means of deliberately targeting agriculture [19]. Entomological warfare may be considered as old as humanity itself [33]: the Roman Empire often catapulted beehives at enemy positions, the Viet Cong set booby traps against American forces with Asian honeybees, and there is even evidence to suggest that Neolithic humans hurled hornet’s nests as makeshift weapons [29].

Use of Stinging Insects

The use of insects as a stinging weapon was extremely rare and usually unplanned during the American Civil War. In the most famous example, at the Battle of Antietam, the 132nd Pennsylvania Volunteers regiment was in the middle of advancing on Confederate lines across a local farm. Unfortunately, as the largely inexperienced soldiers advanced through the farm’s bee yard, an enemy cannon round tore through many of the hives, releasing a cloud of stinging insects across the Union line. The unit nearly disintegrated, and to prevent a rout, it had to be forced into an awkward double-time quick march that left it hideously exposed to Confederate fire. While the regiment suffered heavy casualties, they were ultimately able to continue the Union advance [19].

In one other notable story, one Georgia woman was recorded to have protected her farm against marauding soldiers by setting up several beehive traps: when she saw soldiers attempting to steal honey from her beehives, she yanked a hidden cord, releasing a cloud of stinging insects upon the would-be thieves [19].

While these make for interesting anecdotes, deliberate entomological warfare was extremely limited during the Civil War, with the possible exception of the Confederacy’s struggles with the harlequin bug [19].

The Harlequin Bug

The harlequin bug (*Murgantia histrionica*) is a pest of the southern United States, best defined by its ability to pierce and suck the nutrients from vegetation [34]. It poses a significant threat to agriculture, including cabbage, beans, potatoes, radishes, turnips, asparagus, and even fruit trees [19].

Originally native to Central America [34], the pest appeared in Texas in 1864 and, from there, rapidly spread across the South [35]. For the next several decades, the harlequin bug wreaked economic havoc across the South’s farms [19]. Southerners gave it the disparaging nickname “Sherman-bug” after General Sherman, or even simply a “Lincolnite” after President Lincoln [35]. Rumors reportedly persisted that the pest had been deliberately smuggled into the South by Union spies. The pest’s natural habitat, preferring to stay in the warmer climates of the southern United States, only furthered these rumors [19]. If true, this would mark the first time in history a government was accused of employing insects as a means of agricultural warfare [19].

While the harlequin bug wreaked extensive damage on southern agriculture, there is no evidence whatsoever to support the claim of deliberate entomological warfare [19]. While the harlequin bug did spread rapidly across the American South, during the actual period of the Civil War, it appears to have largely been limited to Texas, limiting any potential efficacy as a biological weapon [36]. Even the appearance of the harlequin bug in the South appears to have been entirely secondary to its natural migration into northern latitudes [19].

## Conclusions

While the current literature is limited, biological warfare in the American Civil War covers a broad number of topics, ranging from Blackburn’s deliberate plot to unleash a yellow fever epidemic across the Union, to Confederate attempts to use the endemic diseases of the South to its advantage, to baseless accusations of entomological warfare. Ultimately, Confederate attempts at biological warfare were stymied by a limited understanding of contemporary diseases, superior Union resources, and the Union’s deliberate efforts to control disease within its ranks. 
